# The new timing in acute care surgery (new TACS) classification: a WSES Delphi consensus study

**DOI:** 10.1186/s13017-023-00499-3

**Published:** 2023-04-28

**Authors:** Belinda De Simone, Yoram Kluger, Ernest E. Moore, Massimo Sartelli, Fikri M. Abu-Zidan, Federico Coccolini, Luca Ansaloni, Giovanni D. Tebala, Salomone Di Saverio, Isidoro Di Carlo, Boris E. Sakakushev, Luigi Bonavina, Michael Sugrue, Joseph M. Galante, Rao Ivatury, Edoardo Picetti, Mircea Chirica, Imtiaz Wani, Miklosh Bala, Ibrahima Sall, Andrew W. Kirkpatrick, Vishal G. Shelat, Emmanouil Pikoulis, Ari Leppäniemi, Edward Tan, Richard P. G. ten Broek, Solomon Gurmu Beka, Andrey Litvin, Elie Chouillard, Raul Coimbra, Yunfeng Cui, Nicola De’ Angelis, Gabriele Sganga, Philip F. Stahel, Vanni Agnoletti, Alessia Rampini, WSES TACS panel of experts, Vishal Shelat, Dimitrios Damaskos, Paolo Carcoforo, Walter L. Biffl, Luigi Bonavina, Andreas Hecker, Isidoro Di Carlo, Fikri M. Abu-Zidan, Joseph M. Galante, Andrew Kirkpatrick, Massimo Sartelli, Edoardo Picetti, Raul Coimbra, Salomone Di Salomone, Zsolt Balogh, Solomon Gurmu Beka, Richard Ten Broek, Georges Velmahos, Boris Sakakushev, Edward Tan, Marco Ceresoli, Osvaldo Chiara, Philip Stahel, Vanni Agnoletti, Emmanouil Pikoulis, Ari Leppaniemi, Ingo Marzi, Giovanni D. Tebala, Ibrahima Sall, Kenji Inaba, Vladimir Khokha, Imtaz Wani, Viktor Reva, Ernest E. Moore, Luca Ansaloni, Mansoor Khan, Adriana Toro, Andrey Litvin, Nicola de’ Angelis, Mark Malangoni, Yoram Kluger, Emanuele Scozzafava, Mircea Chirica, Ian Civil, Ron Maier, Dieter Weber, Massimo Chiarugi, Rao Ivatury, Kjetil Soreide, Gabriele Sganga, Yunfeng Cui, Mario Testini, Francesca Bravi, Ronald V. Maier, Walter L. Biffl, Fausto Catena

**Affiliations:** 1 Department of General and Emergency Surgery, Guastalla Hospital, AUSL Reggio Emilia, Guastalla, Italy; 2 Unit of General and Metabolic Surgery, Clinique de St Louis, Poissy, France; 3 Department of General Surgery, The Rambam Academic Hospital, Haifa, Israel; 4 Ernest E. Moore Shock Trauma Center, University of Colorado, Denver, USA; 5 Department of General Surgery, Macerata Hospital, Macerata, Italy; 6 The Research Office, College of Medicine and Health Sciences, United Arab Emirates University, Al-Ain, United Arab Emirates; 7 Department of General and Trauma Surgery, University Hospital of Pisa, Pisa, Italy; 8 Department of General Surgery, University Hospital of Pavia, Pavia, Italy; 9 U.O.C. Chirurgia Digestiva e d’Urgenza, Azienda Ospedaliera S.Maria, Terni, Italy; 10 Department of General Surgery, Santa Maria del Soccorso Hospital, San Benedetto del Tronto, Ascoli Piceno Italy; 11 Department of Surgical Sciences and Advanced Technologies, University of Catania, Cannizzaro Hospital, Catania, Italy; 12 Research Institute at Medical University Plovdiv/University Hospital St George, Plovdiv, Bulgaria; 13 Division of General and Foregut Surgery, University of Milan, IRCCS Policlinico San Donato, Milan, Italy; 14 Donegal Clinical Research Academy, Letterkenny University Hospital, Letterkenny, Ireland; 15 Division of Trauma and Acute Care Surgery, Department of Surgery, University of California Davis, Sacramento, CA USA; 16 Virginia Commonwealth University, Richmond, VA USA; 17 Department of Anesthesia and Intensive Care, Parma University Hospital, Parma, Italy; 18 Department of Digestive Surgery, Centre Hospitalier Universitaire Grenoble Alpes, La Tronche, France; 19 Government Gousia Hospital, Srinagar, India; 20 Acute Care Surgery and Trauma Unit, Department of General Surgery, Hadassah Medical Center and Faculty of Medicine, Hebrew University of Jerusalem Kiriat Hadassah, Jerusalem, Israel; 21 General Surgery Department, Military Teaching Hospital, Dakar, Senegal; 22 General, Acute Care, Abdominal Wall Reconstruction, and Trauma Surgery, Foothills Medical Centre, Calgary, AB Canada; 23 Department of General Surgery, Tan Tock Seng Hospital, Novena, Singapore; 24 Medical School, National and Kapodistrian University of Athens, (NKUA), Athens, Greece; 25 Abdominal Center, Helsinki University Hospital and University of Helsinki, Helsinki, Finland; 26 Department of Surgery, Radboud University Medical Center, Nijmegen, The Netherlands; 27 Department of General and Trauma Surgery, Ethiopian Air Force Hospital, Bishoftu, Ethiopia; 28 Department of Surgical Disciplines, Regional Clinical Hospital, Immanuel Kant Baltic Federal University, Kaliningrad, Russia; 29 CECORC Research Center, Riverside University Health System, Loma Linda University, Loma Linda, USA; 30 Department of Surgery, Nankai Clinical School of Medicine, Tianjin Nankai Hospital, Tianjin Medical University, Tianjin, China; 31 Colorectal and Digestive Surgery Unit - DIGEST Department, Beaujon University Hospital (AP-HP), Clichy, France; 32 Department of Emergency Surgery, “A. Gemelli Hospital”, Catholic University of Rome, Rome, Italy; 33 Department of Surgery, Brody School of Medicine, East Carolina University, Greenville, USA; 34 Level I Trauma Centre, Bufalini Hospital, Cesena, Italy; 35 Department of General and Emergency Surgery, Level I Trauma Center, Bufalini Hospital, Cesena, Italy; 36 Department of Surgery, University Hospital of Bari, Bari, Italy; 37 Healthcare Administration, Santa Maria Delle Croci Hospital, AUSL Romagna, Ravenna, Italy; 38 Harborview Medical Center, University of Washington, Seattle, WA USA; 39 Department of Emergency and Trauma Surgery, Scripps Clinic Medical Group, La Jolla, CA USA; 40 Department of General Surgery, Level I Trauma Center, Bufalini Hospital, Cesena, eCampus University, CREAS, Ser.In.Ar. Bologna University, Cesena, Italy

**Keywords:** Emergency surgery, Priority, Time to surgery, Delay in surgery, Healthcare system, Classification, Operating room management, Timing in acute care surgery (TACS), Triage, Delphi method

## Abstract

**Background:**

Timely access to the operating room for emergency general surgery (EGS) indications remains a challenge across the globe, largely driven by operating room availability and staffing constraints. The “timing in acute care surgery” (TACS) classification was previously published to introduce a new tool to triage the timely and appropriate access of EGS patients to the operating room. However, the clinical and operational effectiveness of the TACS classification has not been investigated in subsequent validation studies. This study aimed to improve the TACS classification and provide further consensus around the appropriate use of the new TACS classification through a standardized Delphi approach with international experts.

**Methods:**

This is a validation study of the new TACS by a selected international panel of experts using the Delphi method. The TACS questionnaire was designed as a web-based survey. The consensus agreement level was established to be ≥ 75%. The collective consensus agreement was defined as the sum of the percentage of the highest Likert scale levels (4–5) out of all participants. Surgical emergency diseases and correlated clinical scenarios were defined for each of the proposed classes. Subsequent rounds were carried out until a definitive level of consensus was reached. Frequencies and percentages were calculated to determine the degree of agreement for each surgical disease.

**Results:**

Four polling rounds were carried out. The new TACS classification provides 6 colour-code classes correlated to a precise timing to surgery, defined scenarios and surgical condition. The WHITE colour-code class was introduced to rapidly (within a week) reschedule cancelled or postponed surgical procedures. Haemodynamic stability is the main tool to stratify patients for immediate surgery or not in the presence of sepsis/septic shock. Fifty-one surgical diseases were included in the different colour-code classes of priority.

**Conclusion:**

The new TACS classification is a comprehensive, simple, clear and reproducible triage system which can be used to assess the severity of the patient and the surgical disease, to reduce the time to access to the operating room, and to manage the emergency surgical patients within a “safe” timeframe. By including well-defined surgical diseases in the different colour-code classes of priority, validated through a Delphi consensus, the new TACS improves communication among surgeons, between surgeons and anaesthesiologists and decreases conflicts and waste and waiting time in accessing the operating room for emergency surgical patients.

**Graphical Abstract:**

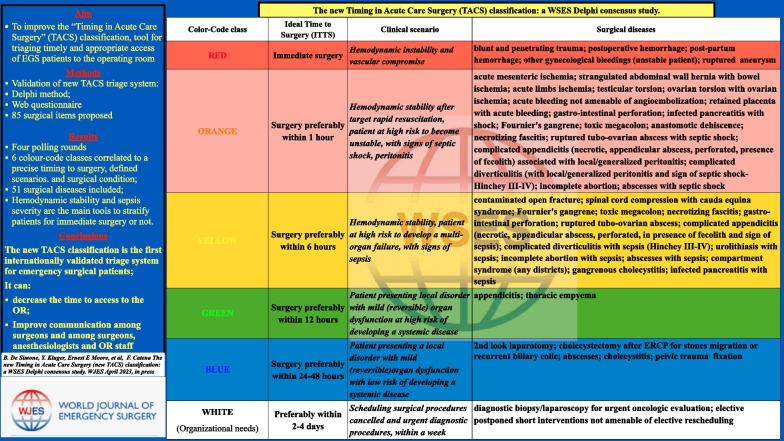

**Supplementary Information:**

The online version contains supplementary material available at 10.1186/s13017-023-00499-3.

## Background

Emergency surgery procedures represent a large and unplanned workload for hospitals worldwide. It is estimated that 28% of the global burden in the emergency setting is surgical [1]. Patients who undergo surgical procedures in the emergency setting are 8 times more likely to die than those undergoing the same procedure electively with added high health systems costs [2]. Early diagnosis and management is the key to improving outcomes and decreasing morbidity, length of hospital stay and mortality in all patients. Delay in the diagnosis and to the operating theatre impacts outcomes, above all in frail, elderly and critically ill patients. Most hospitals have no dedicated operating rooms (OR) for emergency surgery: if the procedure needs to be performed immediately, scheduled procedures must be cancelled or postponed; otherwise, if the patient is haemodynamically stable, the emergency surgical procedure can be performed at the end of the scheduled elective surgical procedures, late in the afternoon or during the night [3].

Performing emergency surgical operations timely and effectively according to the clinical scenario and disease severity is crucial to reduce postoperative complications.

It is well known that not all emergency procedures have the same severity and priority. Without effective control of the operating rooms’ flow, the OR ‘waste time’ and waiting time will increase [3].

An effective triaging system for patients admitted to the Emergency Department (ED) with acute abdomen or trauma is a key step in decision-making to establish the order of priority of the patients due to be operated as emergencies. The triage process includes three factors: the haemodynamic status of the patient, the kind of surgical disease and the severity of sepsis [4].

The importance of providing an effective triage system was highlighted by the experience of the ongoing pandemic. During the first period of the COVID-19 pandemic, due to limited access to operating theatres which were converted into intensive care units for ventilatory mechanical support in severe COVID patients, elective surgical procedures were cancelled and emergency and trauma surgical procedures were managed by emergency/acute care surgeons and anaesthesiologists who had to decide when to apply non-operative management, where to admit the patients to, if to the ICU or the surgical ward, the timing of surgery if needed, and priority of access to OR depending on capacity and availability of resources.

The COVID-19 pandemic had a negative effect on the management of emergency surgical patients, by decreasing the number of patients undergoing emergency surgery and increasing the delay of diagnosis and treatment for those patients [5]. Moreover, all elective procedures that have been cancelled had to be rescheduled. This further increased the delay in the management of emergency patients who were postponed to the end of the elective sessions, in particular in hospitals without a dedicated emergency theatre.

Several different non-validated triage systems were locally implemented, and they often were conflicting, non-reproducible, and non-multidisciplinary and developed by a single, day-by-day designated manager [3].

Different triage systems for emergency surgical patients were nationally implemented and reported in the literature such as the National Confidential Enquiry into Patient Outcome and Death (NCEPOD) ([6] https://www.ncepod.org.uk/classification.html; 2004; NHS-UK), the Groote Schuur Emergency Surgery Triage (GSEST) system [7], the Non-Elective Surgery Triage (NEST) [8].

The key in organizing and managing emergency OR flow is the rapid and safe assessment of the priority and severity of each patient through a simple, clear, validated, comprehensive, reproducible and safe triage system. The timing in acute care surgery (TACS) classification [9], was conceived and proposed by an experienced panel, including international acute care surgeons, to be a valid and accurate tool to establish priority of access of emergency surgical patients to the operating theatres. During the COVID-19 pandemic, the TACS classification was suggested as an effective tool to decrease the delay in the management of patients within limited resources of ORs, healthcare personnel and personal protective equipment [10].

**Fig. 1:**
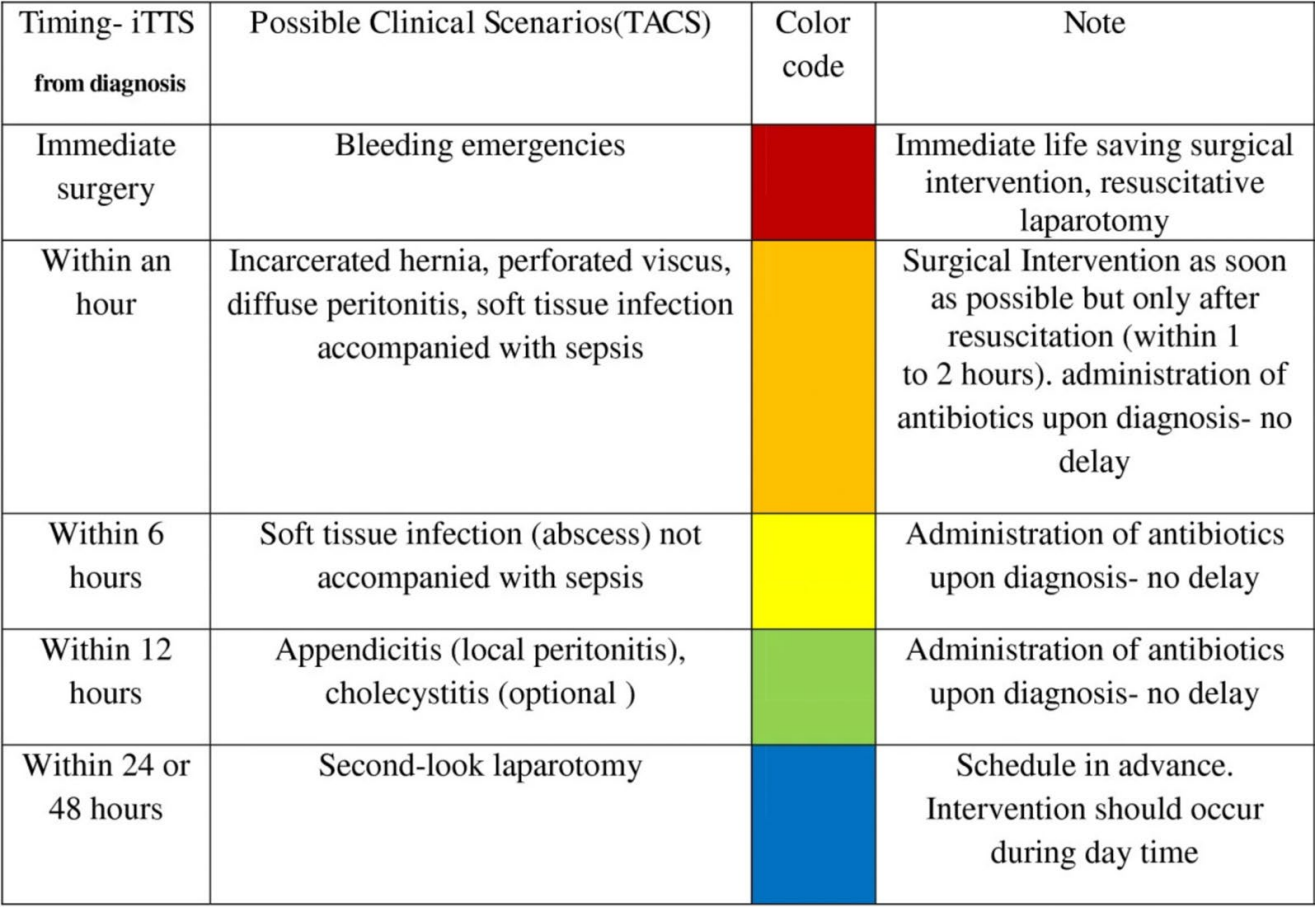
The 2013 TACS classification

The TACS classification is a colour-triage system (Fig. 1) developed according to the “traffic light colour-coding system” in 5 classes of priority/severity/timing for OR admission. These are: (1) RED/immediate surgery; (2) ORANGE/surgery within an hour; (3) YELLOW/surgery within 6 h; (4) GREEN/surgery within 12 h; (5) BLUE/surgery within 24 or 48 h. Every colour-code class is correlated with an ideal time from diagnosis to surgery. This categorizes emergency surgical patients according to the clinical features and the potential adverse effects of delaying the access to OR on outcomes. If there is a change in the patient's condition while waiting, the colour-code class can be changed by the surgeon responsible for the patient's care [11].

Several studies have reported the advantages of the implementation of TACS in clinical practice, mostly in terms of reduced time from admission to the management of surgical patients in the emergency setting [12, 13].

The TACS classification's main limits are that it was not validated after having been proposed and showed a low global implementation in clinical practice, despite its advantages.

The aim of this study is to create an improved TACS classification through a consensus process, to provide a standardized, evidence-based, practical, flexible and generalizable tool to be globally applied in different settings.

## Methods

This is a validation study of the new TACS classification by a selected multidisciplinary expert panel using the Delphi method. It was carried out in 12 months (1st October 2021–22th December 2022) and included two steps: (1) identification of surgical diseases and conditions to be included in the new TACS classification and (2) validation of the new TACS classification.

A review of all the emergency surgical procedures coded according to the International Classification of Diseases (ICD)-10 in a general surgery department and Level I trauma centre was carried out to identify the main causes of admission and the most commonly performed emergency surgical procedures. Eighty-five surgical diseases were identified and listed under the supervision of a multidisciplinary local panel of acute care surgeons and anaesthesiologists, according to evidence-based guidelines of practice (Additional file 1: Material S1). These surgical diseases were included in the new TACS according to colour code, clinical scenarios and ideal timing of surgery.

The modified Delphi method [14] was adopted to validate the new TACS classes, surgical diseases and time to surgery by experts’ consensus. An international panel of 86 surgeons was selected within the scientific community of acute care surgeons and members of the World Society of Emergency Surgery (WSES), with a surgical experience of over 10 years in emergency and trauma surgery, and those who were involved in the previous TACS working group [9]. The panel was multidisciplinary and included acute care surgeons and at least a urologist, a gynaecologist, a neurosurgeon, an otolaryngologist, a maxillo-facial surgeon, a vascular surgeon, and an orthopaedic surgeon.

The TACS questionnaire was designed as a web-based survey according to the Checklist for Reporting Results of Internet E-Surveys (CHERRIES) [15] on a Google Form platform. The questionnaire included 85 closed questions, referring to the Likert scale of agreement 1–5 (1 = strongly disagree–5 = totally agree) divided into 6 colour-code classes. It was sent by mail to the panel of experts for voting. The WSES multidisciplinary expert panel was asked to establish the priority of access to the operating theatre according to the colour-code class, taking into account the severity of diseases. The first Delphi round was opened on 1st October 2021. The collected data were anonymised. The consensus agreement (CA) level was established to be ≥ 75%. The collective consensus agreement (CCA) was defined as the percentage of the sum of the highest Likert scale levels of 4 and 5 out of all participants. Surgical emergency diseases and correlated clinical scenarios were defined for each of the proposed classes. Surgical diseases that had a CA or CCA < 50% were cancelled from the TACS list; those with a CA or CCA between 50 and 75% were sent to the subsequent voting round and presented with a clearer definition and/or a different colour-code class, or cancelled according to the comments of the panellists. Subsequent rounds were carried out until a definitive level of consensus was reached. After each round, responses were analysed to modify the questionnaire for the following round. According to the modified Delphi method, the participation was voluntary and the responses of each panellist remain anonymous. Data were collected and stored in an online database protected by a password known only to the principal investigators. Frequencies and percentages were calculated to determine the degree of agreement for each surgical disease.

### Notes on the use of the new TACS

The new TACS classification triaging system is the result of an extensive review of the literature and a Delphi content validation by a consensus of experts in the field.

The new TACS classification does not represent a standard of practice. It is a suggested plan of care, based on the best available evidence and the consensus of experts, but it does not exclude other approaches as being within the standard of practice. The new TACS classification should be used and tailored by the treating surgeons and individualized for each patient depending on the setting and should not be followed blindly.

## Results

**Fig. 2:**
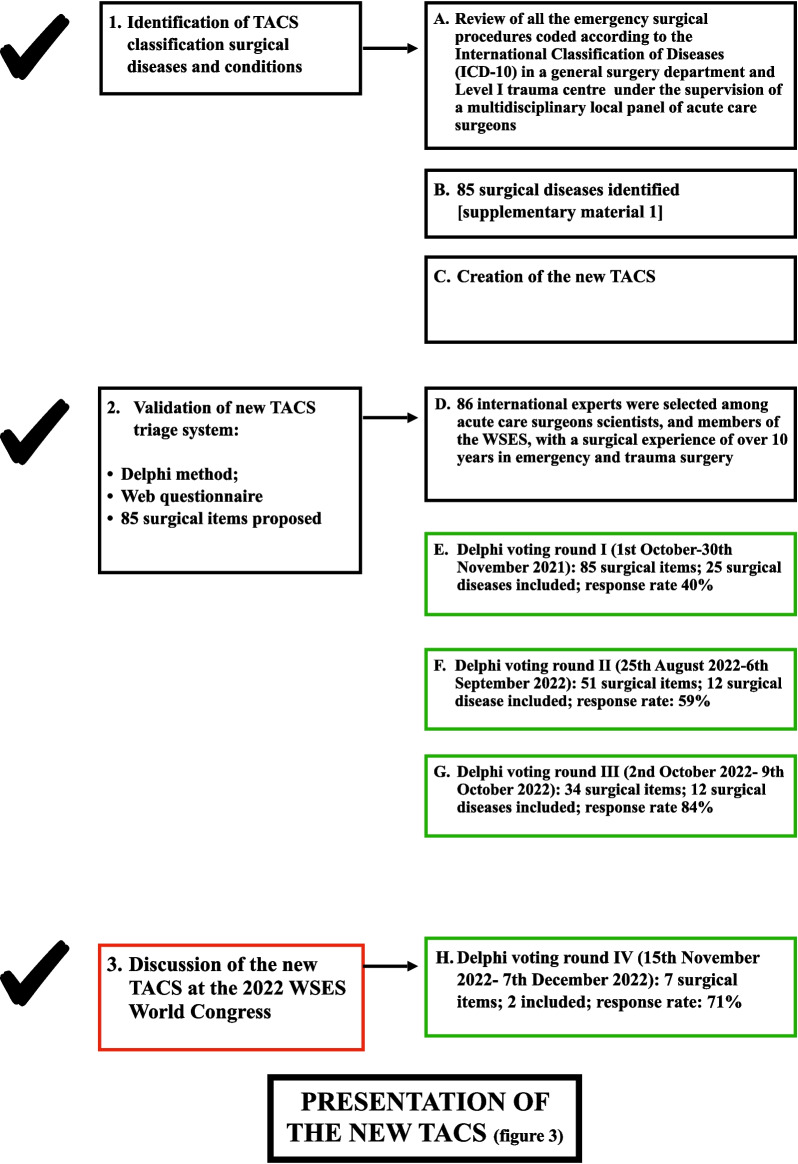
The timeline of the new TACS study

The timeline of the study is summarized in Fig. 2. Items with CA/CCA ≥ 75% were included in the colour-code class proposed using four rounds of voting.

### Delphi round I

The panellists were invited to express their opinion within 2 months (1st October 2021–30th November 2021) by email. The response rate was 40% (35/86). After completing round I, the included surgical diseases are summarized in Additional file 2: Table S1. Eighty-five surgical items were proposed; 6 surgical diseases in unstable patients and those with vascular compromise were included in the RED colour-code class; 14 surgical diseases in patients presenting with haemodynamic stability after target-guided rapid resuscitation, with high risk to become unstable, signs of septic shock or generalized peritonitis were included in the ORANGE colour-code class; 2 surgical conditions in patients with high risk to develop multi-organ failure, with signs of sepsis were included in the YELLOW colour-code class; 1 surgical disease in patients presenting with local disorder and mild organ dysfunction with risk to develop to a systemic disease were included in the GREEN colour-code class; 2 surgical diseases in patients presenting with local disorder and mild organ dysfunction having low risk of developing a systemic disease were included in the BLUE colour-code class. Panellists’ comments and suggestions were summarized. Required modifications were implemented in the TACS questionnaire for the II round of voting.

### Delphi round II

The panellists were invited to express their opinion within 13 days (25th August 2022–6th September 2022) by email. The response rate was 59.3% (51/86). After round II voting was closed, the surgical diseases included in the different colour-code classes are summarized in Additional file 3: Table S2; 51 surgical items were proposed; 1 surgical item was included in the ORANGE colour-code class; 8 surgical conditions were included in the YELLOW colour-code class; 1 surgical disease was included in the GREEN colour-code class; 2 items were included in the WHITE colour-code class that was created to include patients to be postponed due to organization needs, previously cancelled cases to be rescheduled and postponed elective surgical procedures, and diagnostic surgical procedures. Panellists asked for a clearer definition of surgical diseases proposed, haemodynamic status and clinical scenarios. Modifications were implemented in the TACS questionnaire, and several surgical diseases were proposed and added to the III round of voting.

### Delphi round III

The panellists were invited to express their opinion within 8 days (2nd October 2022–9th October 2022) by email, to be in time to present the results of the study at the WSES World Congress in Perth-Australia, which was scheduled for 27–30 October 2022. The response rate was 84% (43/51). Following round III of voting, the surgical diseases included in the different colour-code classes were summarized in Additional file 4: Table S3; 34 surgical items were proposed; 1 surgical disease was included in the RED colour-code class; 5 surgical diseases were included in the ORANGE colour-code class; 4 surgical conditions were included in the YELLOW colour-code class; 1 surgical disease was included in the GREEN colour-code class; 1 surgical disease was included in the BLUE colour-code class. Panellists discussed the results of round III voting at the WSES World Congress 2022. They asked for clearer definitions of the surgical diseases and the physiological status of the patient, omitting surgical diseases that are not generally managed by acute care and emergency surgeons. The TACS questionnaire was modified and prepared for round IV.

### Delphi round IV

**Fig. 3:**
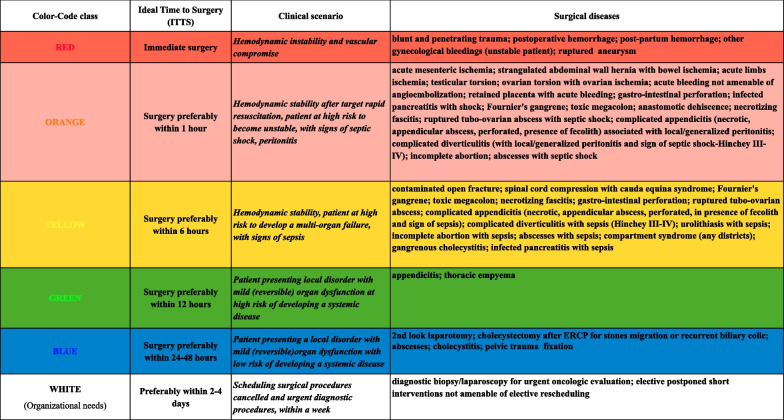
The new TACS classification

The panellists were invited to express their opinion within 22 days (15th November 2022–7th December 2022) by email. The response rate was 71% (32/45). After round IV, the surgical diseases included in the different colour-code classes were summarized in Additional file 5: Table S4; 5 surgical items were proposed; 2 surgical diseases were included in the ORANGE colour-code class. All the items which didn’t reach the CA/CCA were cancelled.In Additional file 6, Table S5 summarizes the final overall outcome of the Delphi process, and Fig. 3 shows the validated new TACS classification.

## Discussion

No validated triaging systems to manage emergency surgical patients in non-austere or war environments are reported in the literature [16].

The COVID-19 pandemic showed that implementing an effective surgical triage is crucial in particular in periods of restricted resources in order to protect frail patients and healthcare workers, and guarantee good use of the available resources while maintaining an acceptable quality of care. After the COVID-19 pandemic, the already stretched healthcare systems are facing the issue of unacceptable long elective waiting lists and the risk of another pandemic. During the early stages of the pandemic, the emergency surgeon was in charge of the decision to operate or delay a surgical procedure or treat non-operatively patients on the basis of national and international specific guidelines for COVID and non-COVID patients, often on the ground of non-evidence-based concepts, with high responsibility [17]. Currently, as “normal” daily practice has returned, the organization of emergency surgery must deal with the necessary rescheduling of previously cancelled elective non-time-dependent surgery but also the issue of restricted staff and lack of dedicated emergency theatres, while considering that outcomes in the emergency setting are time-dependent, in particular in critically ill and frail patients whose survival can be affected by unexpected delays.

An effective triaging system should be clear, simple, transparent, fast and related to the decision‐making process taking into account priorities, the patient’s clinical condition (haemodynamic stability or instability), and the severity of disease (diffuse peritonitis with and without shock; localized peritonitis; bowel obstruction with and without bowel ischaemia; soft tissue infections with and without sepsis) according to evidence-based guidelines [18]. Prioritizing emergency operations by using a risk-stratifying system of different classes is the most effective tool to triage multiple urgent conditions with different severity.

To our knowledge, there are no effective and validated triaging and organization systems to manage the access to the emergency theatre able to decrease the time between admission to the ED and surgical management when needed. The ORSA study [3] showed that most hospitals have no dedicated emergency operating theatre; the emergency operating theatre is not always available; elective surgical procedures were postponed or cancelled to make room for emergency surgery during the day; and the operating rooms flow is managed empirically by the anaesthesiologist often on the basis of local, non-validated and non-reproducible triage systems.

It necessarily follows that the emergency pathway must be reorganized to improve patients' safety, guarantee good use of resources, and decrease costs.

Different triage systems have been recently proposed to improve the flow of emergency surgical patients. To our knowledge, the triaging systems that are largely available are the NCEPOD and TACS classifications. The NCEPOD classification includes 4 categories: immediate, urgent, expedited, and elective. Although largely used in the UK, it is quite inaccurate in defining the right timing to perform a surgical procedure according to the class of priority [6].

The TACS classification was designed to have 5 colour-code classes according to an ideal time for surgery.

It was reported that the traffic light colour-coding system could decrease the delay in operative management. The experience of a single hospital after the introduction of a colour-coding system showed that night emergency surgical procedures significantly decreased from 27.4% before to 23.5% (*p* < 0.001) and red code surgery increased from 45.2 to 62.7% [11].

A prospective study assessed the rate of adherence between the realization of planned surgical urgency and triaging made through the local triage system including 4 classes and based on the surgeon’s perceptions. The study demonstrated that the higher the degree of priority, the greater the chance of the surgery being performed within the required time [12].

A retrospective study in a tertiary hospital which implemented TACS classification in daily routine for 4 surgical specialties with high demand for emergencies demonstrated that the TACS classification significantly improved the timing of surgery in the yellow class and during the night [13].

Moreover, the TACS classification showed more accuracy than NCEPOD in describing a patient’s clinical condition, but the attribution of a patient to a specific class of priority depends on the surgeon’s evaluation which could be questioned by the anaesthesiologist who often regulates the priority of urgent surgical procedures, and surgeons of other specialities, waiting to perform an emergency procedure.

The new TACS classification was conceived to improve clarity in the processes of assigning a patient to a specificity class of severity and reproducibility.

The new TACS provides 6 colour-coding classes correlated with a defined optimal timing of surgery, defined scenarios, patient’s condition and surgical diseases. The WHITE colour-code class was introduced in the new TACS to reschedule cancelled or postponed surgical procedures that need to be rapidly planned, within the week. Haemodynamic stability after resuscitation remains the main tool to stratify patients for immediate surgery or not in the presence of sepsis/septic shock, in defined scenarios such as diffuse peritonitis with and without shock, localized peritonitis, bowel obstruction with and without bowel ischaemia and soft tissues infection with and without sepsis.

By including well-defined surgical diseases in the different colour-coded classes of priority, validated by an expert multidisciplinary panel, the new TACS improves communication among surgeons and anaesthesiologists and decreases conflicts and wasted time.

The attribution of a patient to one or the other colour-coded class makes immediately evident to the anaesthesiologist and the OR team the severity of the condition and the optimal timing of surgery. However, the implementation of an emergency surgery pathway, and therefore the optimization of the workflow in the emergency theatre, is up to the managers of each trust, bearing in mind that there is no acceptable delay for patients when a prolonged delay might lead to life-threatening conditions and poorer outcomes or more invasive surgical treatment and prolonged hospital stay.

Well-defined clinical pathways and timely and appropriate surgical interventions improve outcomes and decrease healthcare systems costs.

### Limitations of the study

To the best of our knowledge, the new TACS is the only available content-validated triage system through a Delphi consensus of experts.

Further prospective multicentric global study is needed to definitely demonstrate the validity and reproducibility of the new TACS in surgical practice and outcomes in terms of timing of OR access and postoperative morbidity and mortality rate.

## Conclusion

An effective and validated triage system for emergency surgery is crucial to reduce the time to theatre for emergency surgical patients and to optimize the workflow also in the elective theatres, in particular in a period of restricted resources.

The new TACS classification is a comprehensive, simple, clear, fast and reproducible triage system. It can be used to stratify the severity of the patient and the surgical disease, to reduce the time to access to the OR and manage the emergency surgical patients within a “safe” timeframe, performing prompt haemodynamic control for unstable patients and adequate surgical source control.

The new TACS classification may improve the management of the emergency operating room workflow in any hospital, decreasing delays in performing emergency surgery, and improving outcomes.

## Supplementary Information

**Additional file 1: Material S1.** List of main emergency surgical diseases.

**Additional file 2: Table S1.** Delphi round I results.

**Additional file 3: Table S2.** Delphi round II results.

**Additional file 4: Table S3.** Delphi round III results.

**Additional file 5: Table S4.** Delphi round IV results.

**Additional file 6: Table S5.** Summary of all Delphi rounds results.

## Data Availability

Supplemental materials are available.

## Abbreviations

OR: Operating room
TACS: Timing in acute care surgery
CA: Consensus agreement
CCA: Collective consensus agreement
WSES: World Society of Emergency Surgery
ED: Emergency Department
ORSA: Operating room surgical activity study
ICU: Intensive care unit
