# Supplementary material for: The new timing in acute care surgery (new TACS) classification: a WSES Delphi consensus study
Source: World J Emerg Surg. 2023 Apr 28;18:32. doi: 10.1186/s13017-023-00499-3 (PMC10147354; doi:10.1186/s13017-023-00499-3)
Supplement: Supplementary file 1 — Additional file 1: Material S1. List of main emergency surgical diseases. [file 13017_2023_499_MOESM1_ESM.docx]

**RED class-immediate surgery surgical items**

1. Blunt and penetrating trauma
2. Postoperative hemorrage
3. GI bleeding (endoscopic hemostasis)
4. Hemorragic corpus luteum
5. Extra-uterine pregnancy
6. Post-partum hémorrage
7. Pelvic trauma external stabilisation
8. Major vascular lesions
9. Pneumothorax
10. Tracheostomy
11. Ruptured abdominal aortic aneurysm
12. Aortic dissection type A
13. Any intracranial conditions with imminent risk of 'coning'
14. Acute extradural haematoma
15. C-section

**ORANGE class-surgery within 1h (stable patient; septic shock/diffuse peritonitis) surgical items**

1. Acute mesenteric ischemia
2. Incarcerated hernia
3. Ischemic limbs ischemia
4. Graft thrombosis
5. Ovarian torsion
6. Testicular torsion
7. Rupture corpus cavernosum
8. Penetrating trauma in stable patient (exploratory laparotomy)
9. Embolization in trauma (stable) patient
10. Retined placenta with acute bleeding (stable patient)
11. Acute metrorrhage
12. Macrohematuria
13. Gastrointestinal perforation: diverticular perforation, peptic ulcer perforation, perforated appendicitis (septic shock/diffuse peritonitis)
14. Fournier gangrene
15. Toxic megacolon
16. Necrotizing hemorrahagic pancreatititis (septic shock)
17. Anastomotic fistula (septic shock/diffuse peritonitis)
18. Ruptured tubo-ovarian abscess
19. Necrotizing fasciitis
20. Intraperitoneal bladder rupture
21. Urolithiasis (septic shock)
22. Bowel obstruction (systemic disease)
23. Retrobulbar hematoma
24. Subdural haematoma
25. Intracerebral haematoma
26. Penetrating injuries
27. Post-operative deterioration
28. Foreign bodies with complete obstruction or batteries
29. Compartment syndrome in any anatomical district
30. Heart and lung transplant

**YELLOW class-surgery within 6hrs (stable patient with sepsis/localized peritonitis) surgical items**

1. Complicated appendicitis (sepsis/localized peritonitis)
2. Cholecystitis (sepsis/ localized peritonitis)
3. Necrotizing hemorrhagic pancreatitis (sepsis/ localized peritonitis)
4. Perianal abscess (sepsis)
5. Diverticulitis (sepsis/ localized peritonitis)
6. Cholangitis  (sepsis)  (biliary tract drainage)
7. Abscess (sepsis)  (percutaneous drainage)
8. Acute infectious arthritis (sepsis)
9. Contaminated open fracture
10. Urolithiasis (sepsis)
11. Ureteral stent removal (sepsis)
12. Nephrostomy removal (sepsis)
13. Incomplete abortion (sepsis)
14. Bowel obstruction (moderate organ dysfunction)
15. Anastomotic fistula (moderate organ dysfunction)
16. Pneumothorax (moderate organ dysfunction)
17. Urinary retention (cistostomy) (moderate organ dysfunction)
18. Epistaxis
19. Post traumatic pseudoaneurysm (embolization)
20. Spinal cord compression by trauma or tumours
21. Intracranial tumours causing critical raised intracranial pressure
22. Skull fractures
23. Blocked shunt
24. Liver transplant

**GREEN class-surgery within 12 hrs (stable patient) surgical items:**

1. Appendicitis (infection)
2. Cholecystitis (infection)
3. Perianal abscess (infection)
4. Thoracic empyema (infection)
5. Cholangytis (ERCP)
6. Cholecystitis (AXIOS stent)
7. Peritonsillar abscess (infection)
8. Obstructed hernia (mild organ dysfunction)
9. Bowel obstruction (mild organ dysfunction)
10. Caustic ingestion (mild organ dysfunction)
11. Ureteral lesion (mild organ dysfunction)
12. Urinary fistula (mild organ dysfunction)
13. Hydronephrosis (stent position) (mild organ dysfunction)
14. Urolithiasis (acute kidney injury)
15. Cerebral abscess
16. Pituitary apoplexy
17. Hemorragic corpus luteum (free abdominal fluid)
18. Kidney and pancreatic transplant
19. Foreign body without complete obstruction

**BLUE class-surgery within 24/48 hrs surgical items:**

1. Second-look laparotomy
2. Sequential cholecystectomy (after ERCP for gallstone migration)
3. Uncomplicated appendicitis
4. Lower extremity ulcer medication
5. Osteomyelitis (amputation)
6. Arteriovenous fistulae for haemodialysis
7. Symptomatic carotid artery stenosis (crescendo TIA, stroke in evolution)
8. Symptomatic abdominal aortic aneurysm (failure of medical therapy)
9. II R Symptomatic aortic dissection type B (failure of medical therapy)
10. GI bleeding (endoscopy)
11. Bone fractures
12. Tendon lesion
13. Stable pelvic trauma
14. Maxillofacial fracture
15. Tracheostomy for prolonged intubation
16. Hydrocephalus
17. Chronic subarachnoid haematoma
18. Aneurysms AVMs
19. Herniation with impending cauda equina syndrome
20. Posterior fossa mass lesions
21. Burn debridement/graft
22. Urolithiasis after medical failure
23. Osteitis (dental extraction)
24. Foreign gastric body

**ORGANIZATIVE NEED surgical items:**

1. Elective cancelled surgical procedures
2. Diagnostic biopsy/diagnostic laparoscopy
3. PEG placement
4. Stent/nephrostomy removal
