# Supplementary material for: The new timing in acute care surgery (new TACS) classification: a WSES Delphi consensus study
Source: World J Emerg Surg. 2023 Apr 28;18:32. doi: 10.1186/s13017-023-00499-3 (PMC10147354; doi:10.1186/s13017-023-00499-3)
Supplement: Supplementary file 2 — Additional file 2: Table S1. Delphi round I results. [file 13017_2023_499_MOESM2_ESM.pdf]

		I ROUND DELPHI				Tota l	
**COLOR CODE**	**Surgical disease/ conditions**	**Likert scale 4 (near totally agree)**	**%**	**Likert scale 5 (totally agree)**	**%**		**Decision**
**RED CODE- immediate surgery**	**blunt and penetrating trauma**	4/35	11.4	29/35	82.9	>90	**included**
	**postoperative hemorrhage**	5/35	14.3	27/35	77.1	>90	**included**
	**GI bleeding (endoscopic hemostasis)**	11/35	31.4	18/35	51.4	82.8	**included**
	Hemorragic corpus luteum	7/35	20	16/35	45.7	65.7	**II**
	**ruptured aortic aneurysm**	4/35	11.4	30/35	85.7	>90	**included**
	**Tension pneumothorax**	7/35	20	25/35	71.4	>90	**included**

	**Post partum hemorrage**	5/35	14.3	27/35	77.1	>90	**Included**
	**Extra-uterine pregnancy**	6/35	17.1	24/35	68.6	85.7	**Included**
	**Pelvic trauma stabilisation**	9/35	25.7	18/35	51.4	77.1	**Included**
	**Major vascular lesions**	2/35	5.7	30/35	85.7	>90	**Included**
	Tracheostomy	7/35	20	21/35	60	80	**?**
	**Aortic dissection type A**	6/35	17.1	22/35	62.9	80	**Included**
	**Any cranial conditions with risk of coning**	8/35	22.9	23/35	65.7	88.6	**Included**
	**Acute Extradural hematoma**	8/35	22.9	24/35	68.6	>90	**Included**
	**Cesarean section**	10/35	28.6	17/35	48.6	>90	**Included**

**ORANGE CODE-surgery within 1 hour**	**acute mesenteric ischemia**	7/35	20	25/35	71.4	>90	**included**
	**Incarcerated/ strangulated hernia**	13/35	37.1	14/35	40	77.1	**Included**
	**acute limbs ischemia**	7/35	20	25/35	71.4	>90	**included**
	**graft thrombosis**	11/35	31.4	17/35	48.6	80	**included**
	**testicular torsion**	7/35	20	25/35	71.4	>90	**included**
	ovarian torsion	9/35	25.7	17/35	48.6	74.3	**II ?**
	**rupture of corpus cavernosum**	15/35	42.9	12/35	34.3	77.2	**Included**
	**Penetrating trauma in stable patient (exploratory laparotomy)**	15/35	42.9	13/35	37.1	80	**Included**

	Embolization in stable trauma patient	12/35	34.3	14/35	40	74.3	**?**
	**retained placenta with acute bleeding**	10/35	28.6	17/35	48.6	77.2	**included**
	Acute methrorrage	12/35	34.3	10/35	28.6	62.9	**II**
	Macrohematuria	7/35	20	5/35	14.3	34.3	**EXCLUDED <50**
**Patients with septic shock**	**GI perforation**	8/35	22.9	21/35	60	82.9	**included**
	Infected necrotizing haemorrhage pancreatitis	7/35	20	13/35	37.1	57.1	**II ?**
	Urolithiasis with septic shock	10/35	28.6	11/35	31.4	60	**II**
	**Fournier's gangrene**	10/35	28.6	22/35	62.9	>90	**included**
	**toxic megacolon**	10/35	28.6	21/35	60	88.6	**included**

**anastomotic fis tula with septic shock/diffuse peritonitis**	10/35	28.6	17/35	48.6	77.2	**included**
**necrotizing fascitis**	7/35	20	24/35	68.6	88.6	**included**
ruptured tubo-ovarian abscess	12/35	34.3	10/35	28.6	62.9	**II**
Intraperitoneal bladder rupture	6/35	17.1	13/35	37.1	54.2	**II**
Bowel obstruction (systemic disease)	13/35	37.1	13/35	37.1	74.2	**II**
Retrobulbar hematoma	9/35	25.7	17/35	48.6	74.3	**II**
Subdural hematoma	6/35	17.1	18/35	51.4	68.5	**II**
Intracerebral hematoma	9/35	25.7	15/35	42.9	68.6	**II**

	**Foreign GI bodies with complete obstruction or batteries (endoscopy)**	13/35	37.1	14/35	40	77.1	**Included**
Other conditions	**compartment syndrome (any districts)**	6/35	17.1	26/35	74.3	>90	**Included**
	**heart lung transplants**	6/35	17.1	23/35	65.7	82.8	**included**
**YELLOW- surgery within 3/6 hrs**	**contaminated open fracture**	9/35	25.7	19/35	54.3	77.2	**Included**
	Appendicitis with localised peritonitis/ sepsis	8/35	22.9	18/35	51.4	74.3	**II**
	Cholecystitis (localised peritonitis/ sepsis)	9/35	25.7	14/35	40	65.7	**II**

Necrotising hemorrhagic pancreatitis with sepsis/localized peritonitis	9/35	25.7	11/35	31.4	57.1	**II**
Perianal abscess with sepsis	12/35	34.3	10/35	28.6	62.9	**II**
Diverticulitis with sepsis/ localized peritonitis	14/35	40	11/35	31.4	71.4	**II**
**spinal cord compression with cauda equina syndrome**	15/35	42.9	16/35	45.7	88.6	**Included**
**increasing intracranial pressure**	16/35	45.7	15/35	42.9	88.6	**Included**
**anastomotic fis tula with septic shock/diffuse peritonitis**	17/35	48.6	10/35	28.6	77.6	**Included**

Other conditions	**Pneumothorax**	14/35	40	16/35	45.7	85.7	**Included**
	**Bowel obstruction**	17/35	48.6	11/35	31.4	80	**Included**
	Urolithiasis	13/35	37.1	12/35	34.3	71.4	**II**
	Incomplete abortion with sepsis	14/35	40	12/35	34.3	74.3	**II**
	**liver transplants**	9/35	25.7	19/35	54.3		**included**
	Embolization of post-traumatic pseudoaneurys m	11/35	31.4	14/35	40	71.4	**II**
**GREEN-surgery within 12 hrs**	bowel obstruction	6/35	17.1	16/35	45.7	62.8	**II**
	Perianal abscess	12/35	34.3	13/35	37.1	71.4	**II**
	Cholecystitis	8/35	22.9	12/35	34.3	57.2	**II**

	appendicitis	10/35	28.6	16/35	45.7	74.3	**II**
	**Thoracic empyema**	14/35	40	13/35	37.1	77.1	**included**
	incarcerated hernia with obstruction	7/35	20	14/35	40	60	**II**
	Urinary fistula	8/35	22.9	8/35	22.9	45.8	**II**
	Hydronephrosis (stent placement)	10/35	28.6	12/35	34.3	62.9	**II**
	Urolithiasis with acute kidney injury	10/35	28.6	12/35	34.3	62.9	**II**
	kidney and pancreatic transplant	10/35	28.6	15/35	42.9	71.5	**II**
	foreign body without obstruction	11/35	31.4	13/35	37.1	68.5	**II**

**BLUE CODE- surgery within 24-48hrs**	**2nd look laparotomy**	8/35	22.9	22/35	62.9	85.8	**included**
	**cholecystectomy after ERCP for stones migration or recurrent biliary colic**	9/35	25.7	18/35	51.4	77.1	**included**
	Uncomplicated appendicitis	7/35	20	16/35	45.7	65.7	**II**
	Amputation for osteomyelitis	12/35	34.3	11/35	31.4	65.7	**II**
	A-V fistulae for hemodialysis	9/35	25.7	11/35	31.4	57.1	**II**
	Symptomatic carotid artery stenosis, TIA, STROKE in evolution	9/35	25.7	13/35	37.1	62.8	**II**

	Symptomatic AAA after medical treatment failure	9/35	25.7	15/35	42.9	68.6	**II**
	Symptomatic aortic dissection type B	9/35	25.7	16/35	45.7	71.4	**II**
	Bones fracture	11/35	31.4	13/35	37.1	68.5	**II**
	pelvic trauma fixation	15/35	42.9	10/35	28.6	71.5	**II**
	Maxillofacial fractures	9/35	25.7	15/35	42.9	68.6	**II**
	Urolithiasis	13/35	37.1	9/35	25.7	62.8	**II**
**WHITE CODE-ORGANIZATIVE NEED**	Elective postponed interventions	10/35	28.6	13/35	37.1	65.7	**II**
	diagnostic biopsy/ laparoscopy	11/35	31.4	11/35	31.4	62.8	**II**
	PEG placement (endoscopy)	9/35	25.7	10/35	28.6	54.3	**Cancelled**
