# Supplementary material for: The new timing in acute care surgery (new TACS) classification: a WSES Delphi consensus study
Source: World J Emerg Surg. 2023 Apr 28;18:32. doi: 10.1186/s13017-023-00499-3 (PMC10147354; doi:10.1186/s13017-023-00499-3)
Supplement: Supplementary file 3 — Additional file 3: Table S2. Delphi round II results. [file 13017_2023_499_MOESM3_ESM.pdf]

			II ROUND DELPHI					
**COLOR CODE**	**Surgical disease/ conditions**	**Decision I ROUND**	**Likert scale 4 (near totally agree)**	**%**	**Likert scale 5 (totally agree)**	**%**	**Likert scale 5/4+5**	**Decision**
**RED CODE- immediate surgery**	**blunt and penetrating trauma**	**INCLUDED**	-		-	-		-
	**postoperative hemorrhage**	**INCLUDED**	-	-	-	-		-
	**post-partum hemorrhage**	**INCLUDED**	-	-	-	-		-
	**ruptured aneurysm**	**INCLUDED**	-	-	-	-		-
	**Tension pneumothorax**	**INCLUDED**	-	-	-	-		-
**ORANGE CODE-surgery within 1 hrs**	**acute mesenteric ischemia**	**INCLUDED**	-	-	-	-		-

	**acute limbs ischemia**	**INCLUDED**	-	-	-	-		**-**
	**graft thrombosis**	**INCLUDED**	-	-	-	-		**-**
	**testicular torsion**	**INCLUDED**	-	-	-	-		**-**
	**retained placenta with acute bleeding**	**INCLUDED**	-	-	-	-		**-**
**Patients with septic shock**	**GI perforation**	**INCLUDED**	-	-	-	-		**-**
	**Infected necrotizing haemorrhage pancreatitis**	**II ROUND**	8/51	15.7	22/51	43	58.7	**Not included, but why?**<br>**Better definition ? III**
	**Urolithiasis complicated by hydronephrosi s**	**II ROUND**	14/51	27.5	22/51	43	70.5	**Not included, better definition ? III**
	**Fournier's gangrene**	**INCLUDED**	-	-	-	-		

	toxic megacolon	INCLUDED	-	-	-	-		
	anastomotic fis tula	INCLUDED	-	-	-	-		
	necrotizing fascitis	INCLUDED	-	-	-	-		
	ruptured tubo-ovarian abscess	II ROUND	12/51	23.5	24/51	47	70.5	Not included, why if patient is in septic shock?<br>Better definition<br>III
	Incomplete abortion	II ROUND	13/51	25.5	24/51	47	72.5	III
	Perianal abscess	II ROUND	12/51	23.5	26/51	51	74.5	III
Other conditions	pneumothorax	INCLUDED	-	-	-			
	heart lung transplants	INCLUDED	-	-	-			

**YELLOW- surgery within 3/6 hrs**	**Gangrenous cholecystitis**	**II ROUND (added)**	15/51	29.4	26/51	56.9	86.3	**INCLUDED**
	**spinal cord compression with cauda equina syndrome**	**INCLUDED**	-	-	-			
**Patients with sepsis associated with:**	**Fournier's gangrene**	**II ROUND**	6/51	11.8	35/51	68.6	**80.4**	**INCLUDED**
	**toxic megacolon**	**II ROUND**	15/51	29.4	31/51	60.8	**90.2**	**INCLUDED**
	**anastomotic fis tula with local peritonitis**	**II ROUND**	18/51	35.3	17/51	33	68.3	**III**
	**necrotizing fascitis**	**II ROUND**	8/51	15.7	33/51	64.7	**80.4**	**INCLUDED**
	**GI perforation**	**II ROUND**	6/51	11.8	39/51	76.5	**88.3**	**INCLUDED**
	**Ruptured tubo-ovarian abscess**	**II ROUND**	16/51	31.4	26/51	51	**82.4**	**INCLUDED**

	**complicated appendicitis**	**II ROUND**	16/51	31.4	21/51	41.2	72.6	**Better definition?III**
	**Complicated diverticulitis**	**II ROUND**	16/51	31.4	17/51	33.3	64.7	**Better definition?III**
	**Urolithiasis with sepsis**	**II ROUND**	13/51	25.5	17/51	33.3	58.8	**Better definition?III**
	**Incomplete abortion**	**II ROUND**	15/51	29.4	27/51	52.9	**82.3**	**INCLUDED**
	**Perianal abscess with sepsis**	**II ROUND**	17/51	33.3	20/51	39.2	72.5	**Better definition?III**
Other conditions	**Intraperitoneal bladder rupture**	**II ROUND**	18/51	35.3	19/51	37.3	72.3	**Why? Patient in sepsis?**<br>**III**
	**compartment syndrome (any districts)**	**II ROUND**	8/51	15.7	34/51	66.7	**82.4**	**INCLUDED**

	**foreign body with GUT obstruction (including endoscopy)**	**II ROUND**	18/51	35.3	15/51	29.4	64.7	**Better definition? III**
	**liver transplant**	**INCLUDED**	-	-	-	-		-
	**Embolization of post-traumatic pseudoaneurysm**	**II ROUND added**	16/51	31.4	21/51	41.2	72.6	**III**
**GREEN-surgery within 12 hrs**	**bowel/ intestinal obstruction**	**II ROUND**	15/51	29.4	19/51	37.3	66.7	**? III**
	**Perianal abscess**	**II ROUND**	12/51	23.5	21/51	41.2	64.7	**? III**
	**Cholecystitis**	**II ROUND**	13/51	25.5	18/51	35.3	60.8	**? III**

**Appendicitis with systemic signs of infection**	**II ROUND**	11/51	21.6	32/51	62.7	**84.3**	**INCLUDED**
**Thoracic empyema**	**INCLUDED**	-	-	-			**-**
**incarcerated hernia with obstruction**	**II ROUND**	9/51	17.6	28/51	54.9	72.5	**? III**
**Urinary fistula**	**II ROUND**	9/51	17.6	11/51	21.6	39.2	**Cancelled**
**Hydronephrosis (stent placement)**	**II ROUND**	13/51	25.5	16/51	31.4	56.9	**?III**
**Urolithiasis with acute kidney injury**	**II ROUND**	13/51	25.5	16/51	31.4	56.9	**? III**
**kidney and pancreatic transplant**	**II ROUND**	13/51	25.5	21/51	41.2	66.7	**? III**

	**foreign body without obstruction**	**II ROUND**	9/51	17.6	12/51	23. 5	41.1	**Cancell ed**
**BLUE CODE- surgery within 24-48hrs**	**2nd look laparotomy**	**INCLUDED**	-	-	-	-		**-**
	**cholecystectomy after ERCP for stones migration or recurrent biliary colic**	**INCLUDED**	-	-	-	-		**-**
	**Uncomplicated appendicitis**	**II ROUND**	7/51	13.7	31/51	60. 8	74.5	**? III**
	**Amputation for osteomyelitis**	**II ROUND**	6/51	11.8	30/51	58. 8	70.6	**? III**
	**A-V fistulae for hemodialysis**	**II ROUND**	8/51	15.7	28/51	54. 9	70.6	**? III**

	**Symptomatic carotid artery stenosis, TIA, STROKE in evolution**	**II ROUND**	8/51	15.7	19/51	37.3	53	**? III**
	**Symptomatic AAA after medical treatment failure**	**II ROUND**	15/51	29.4	21/51	41.2	70.6	**? III**
	**Symptomatic aortic dissection type B**	**II ROUND**	9/51	17.6	21/51	41.2	58.8	**? III**
	**Bones fracture**	**II ROUND**	13/51	25.5	22/51	43.1	68.6	**? III**
	**pelvic trauma fixation**	**II ROUND**	13/51	25.5	27/51	52.9	**78.4**	**INCLUDED**
	**Maxillofacial fractures**	**II ROUND**	12/51	23.5	25/51	49	72.5	**III**
	**Urolithiasis after medical treatment failure**	**II ROUND**	13/51	25.5	19/51	37.3	62.8	**? III**

**ORGANIZATIVE NEED**	**Elective postponed interventions**	**II ROUND**	12/51	23.5	28/51	54.9	**82.9**	**INCLUDED**
	**diagnostic biopsy/ laparoscopy**	**II ROUND**	9/51	17.6	30/51	58.8	**76.4**	**INCLUDED**
