# Supplementary material for: The new timing in acute care surgery (new TACS) classification: a WSES Delphi consensus study
Source: World J Emerg Surg. 2023 Apr 28;18:32. doi: 10.1186/s13017-023-00499-3 (PMC10147354; doi:10.1186/s13017-023-00499-3)
Supplement: Supplementary file 4 — Additional file 4: Table S3. Delphi round III results. [file 13017_2023_499_MOESM4_ESM.pdf]

III ROUND DELPHI							
**COLOR CODE CLASS**	**SURGICAL DISEASE**	**Likert scale 4 (near totally agree)**	**%**	**Likert scale 5 (totally agree)**	**%**	**Reached Consensus Agreement (>=75%)**	**Decision**
***RED class-Immediate* - unstable patient**	**Gynecological bleeding with unstable patient**	6/43	**14**	35/43	**81.4**	**Y**	**Included**
ORANGE class-Within 1 hour from diagnosis- but as soon as possible, patient stable after target resuscitation but at risk of MOF	**Acute bleeding non amenable of angio-embolization**	16/43	**37.2**	24/43	**55.8**	**Y**	**Included**
	Ovarian torsion with unstable patient	19/43	**44.2**	13/43	**30.2**	N	IV round
	Strangulated (groin, incisional, ventral) hernia	12/43	**27.9**	18/43	**41.9**	N	IV round
	Gynecological bleeding in stable patient after resuscitation	19/43	**44.2**	10/43	**23.3**	N	IV round
	Ruptured tubo-ovarian abscess with septic shock	16/43	**37.2**	15/43	**34.9**	N	IV round

	**Complicated appendicitis (necrotic, appendicular abscess, perforated, presence of fecolith) associated with local/generalized peritonitis and signs of septic shock**	12/43	**27.9**	21/43	**48.8**	**Y**	**Included**
	**Complicated diverticulitis with local/generalized peritonitis and signs of septic shock (Hinchey III-IV)**	11/43	**25.6**	24/43	**55.8**	**Y**	**Included**
	**Perianal abscess with septic shock**	12/43	**27.9**	21/43	**48.8**	**Y**	**Included**
	Infected necrotizing hemorragic pancreatitis with signs of septic shock	11/43	**25.6**	17/43	**39.5**	N	
	Subdural hematoma	15/43	**34.9**	15/43	**34.9**	N	Cancelled
YELLOW class-Within 6 hours from diagnosis-stable patient with signs of sepsis	Anastomotic intestinal fistula/insufficiency in patient presenting sepsis signs	15/43	**34.9**	16/43	**37.2**	N	Modified definition; IV round
	Increasing intracranial pressure conditions	12/43	**27.9**	19/43	**44.2**	N	Cancelled
	**Complicated diverticulitis with sepsis (Hinchey III-IV)**	15/43	**34.9**	23/43	**53.5**	**Y**	**Included**

	**Complicated appendicitis (necrotic, appendicular abscess, perforated, presence of fecolith) associated with sepsis**	16/43	**37.2**	21/43	**48.8**	**Y**	**Included**
	Angioembolization of post splenic trauma pseudoaneurysm	10/43	**23.3**	14/43	**32.6**	N	Cancelled
	Foreign body with GUT obstruction (including endoscopy)	7/43	**16.3**	19/43	**44.2**	N	Cancelled
	Intraperitoneal bladder rupture	11/43	**25.6**	18/43	**41.9**	N	Cancelled
	Kidney transplant	8/43	**18.6**	12/43	**27.9**	N	Cancelled
	**Perianal abscess with sepsis**	23/43	**53.5**	13/43	**30.2**	Y	**Included**
	**Urolithiasis with sepsis**	21/43	**48.8**	13/43	**30.2**	**Y**	**Included**
	Urolithiasis with high risk of kidney injury	12/43	**27.9**	19/43	**44.2**	N	Modified definition; IV round

GREEN class-Within 12 hours from diagnosis- *stable patient with moderate risk of presenting hemodynamic instability and organs failure; admitted in surgical department for prompt medical treatment and monitoring*	Incarcerated abdominal wall hernia with intestinal obstruction	14/43	**32.6**	13/43	**30.2**	N	Modified; IV round
	Bilateral hydronephrosis	9/43	**20.9**	13/43	**30.2**	N	Cancelled
BLUE class-Within 24 or 48 hour from diagnosis*; stable patient with low risk of presenting organs failure, admitted in surgical department for clinical monitoring and medical treatment*	Bowel/intestinal obstruction after medical treatment failure	12/43	**27.9**	18/43	**41.9**	N	modified; IV round
	Perianal abscess	15/43	**34.9**	16/43	**37.2**	N	Modified; IV round
	**Cholecystitis**	10/43	**23.3**	24/43	**55.8**	**Y**	**Included**
	Urinary fistula	5/43	**11.6**	16/43	**37.2**	N	Cancelled
	Foreign body without intestinal obstruction	7/43	**16.3**	23/43	**53.5**	N	Cancelled
	Amputation due to osteomyelitis	14/43	**32.6**	16/43	**37.2**	N	Cancelled

	Acute appendicitis without peritoneal fluid	6/43	**14**	22/43	**51.2**	N	Modified; IV round
	A-V fistula for hemodialiysis	10/43	**23.3**	12/43	**27.9**	N	Cancelled
	Complicated bones fractures (displaced, complex)	13/43	**30.2**	15/43	**34.9**	N	Cancelled
	Maxillo facial fractures	8/43	**18.6**	17/43	**39.5**	N	Cancelled
