# Supplementary material for: The new timing in acute care surgery (new TACS) classification: a WSES Delphi consensus study
Source: World J Emerg Surg. 2023 Apr 28;18:32. doi: 10.1186/s13017-023-00499-3 (PMC10147354; doi:10.1186/s13017-023-00499-3)
Supplement: Supplementary file 5 — Additional file 5: Table S4. Delphi round IV results. [file 13017_2023_499_MOESM5_ESM.pdf]

Class Color-Code	Surgical Diseases	Likert 4 scale (n.)	%	Likert 5 scale (n.)	%	Consensus agreement (Likert 4+5) %	Decision
RED CODE-immediate surgery-patient presenting hemodynamic instability-vascular compromise	Completed						
ORANGE class-Within 1 hour from diagnosis- but as soon as possible, patient stable after target resuscitation, at risk of MOF	Strangulated AW hernia with bowel ischemia	5/32	15.6	21/32	65.6	(5+21) 81.25	Included in the ORANGE class
	Ovarian torsion with ovarian ischemia	11/32	34.4	16/32	50	(11+16) 84.3	Included in the ORANGE class
	Urolithiasis with septic shock	8/32	25.6	12/32	38.7	(8+12) 62.5	Removed
YELLOW class-Within 3/6 hours from diagnosis-stable patient with signs of sepsis, at high risk to develop a multi-organ failure	Completed						

Class Color-Code	Surgical Diseases	Likert 4 scale (n.)	%	Likert 5 scale (n.)	%	Consensus agreement (Likert 4+5) %	Decision
GREEN class-Within 12 hours from diagnosis-stable patient with moderate risk of presenting hemodynamic instability and organs failure; admitted in surgical department for prompt medical treatment and monitoring	Completed						
BLUE class-Within 24/48 hour from diagnosis; stable patient with low risk of presenting organs failure, admitted in surgical department for clinical monitoring and medical treatment	Incarcerated AW hernia with obstruction	9/32	28.1	10/32	31.3	(9+10) 59.3	Removed
	Bowel/intestinal obstruction after medical treatment failure	6/32	18.8	10/32	31.3	(6+10) 50	Removed
