# Supplementary material for: The new timing in acute care surgery (new TACS) classification: a WSES Delphi consensus study
Source: World J Emerg Surg. 2023 Apr 28;18:32. doi: 10.1186/s13017-023-00499-3 (PMC10147354; doi:10.1186/s13017-023-00499-3)
Supplement: Supplementary file 6 — Additional file 6: Table S5. Summary of all Delphi rounds results. [file 13017_2023_499_MOESM6_ESM.pdf]

COLOR CODE	Surgical disease/conditions	I Round Likert scale 4-5 (%)	CCA (%)	Decision	II Round (%)	CCA	Decision	III Round Likert scale 4-5 (%)	CCA	Decision	IV Round	CCA	Decision
**RED CODE- immediate surgery unstable patients**	**Blunt and penetrating trauma**	82.9 (LS 5)<br>11.4 (LS 4)	>90	Included	-		-	-	-	-	-		
	**Postoperative hemorrhage**	77.1(LS 5)<br>14.3 (LS 4)	>90	Included	-	-	-	-	-	-	-		
	**Post-partum hemorrhage**	77.1(Ls 5)<br>14.3(Ls 4)	>90	Included	-	-	-	-	-	-	-		
	**Other gynecological bleedings (unstable patient)**	-	-	-	-	-	-	14 (Ls 4)<br>81 (Ls 5)	>90	Included			
	**Ruptured aneurysm;**	85.7 (5)<br>11.4 (4)	>90	Included	-	-	-	-	-	-			
	**Tension pneumothorax**	71(5)<br>20(4)	>90	Included	-	-	-	-	-	-			

**ORANGE CODE-surgery within 1 hour**	**Acute mesenteric ischemia**	71.4(5) 20(4)	>90	Included	-	-	-	-	-	-			
	Incarcerated hernia	37.1(4) 40 (5)	77	Included with modifications	-	-	Improving definition for III round (strangulated groin, incisional, ventral hernia)	27.9(4) 41.9(5)	69.8	Accepted with modifications for IV Round (**strangulated AW with bowel ischemia**)	15.6 (4) 65.6 (5)	81.2	Included
	**Acute limbs ischemia**	71(5) 20(4)	>90	Included	-	-	-	-	-	-			
	**Graft thrombosis**	48(5) 31(4)	79	Cancelled after panelists suggestions	-	-	-	-	-	-			
	**Testicular torsion**	71(5) 20(4)	>90	Included	-	-	-	-	-	-			

	**Ovarian torsion**	48(5)<br>25(4)	73	II Round	44.2 (Ls 4)<br>30.2 (Ls 5)	74	III Round	44.2 (Ls 4)<br>30.2 (Ls 5)	74	Accepted with modification for IV Round (**Ovarian torsion with ovarian ischemia**)	34.4(4)<br>50(5)	84.4	Included
	**Acute bleeding not amenable of angioembolization**	-	-	-	-	-	III Round	37.2 (Ls 4)<br>55.8 (Ls 5)	93	Included			
	**Retained placenta with acute bleeding**	48(5)<br>28(4)	76	Included	-	-	-	-	-	-			

	Other gynecological bleeding(stable patient after resuscitation)	-	-	-	-	-	III Round	44.2 (Ls 4)<br>23.3 (Ls 5)	67.5	**Cancelled**			
**Patients with septic shock**	**GI perforation**	60(5)<br>23(4)	83	included	-	-	-	-	-	-			
	**Infected necrotizing haemorrhage pancreatitis**	37(5)<br>20(4)	57	II Round	43(5)<br>15(4)	58	III Round	25.6 (Ls 4)<br>55.8 (Ls 5)	81.4	Included			
	Urolithiasis	31(5)<br>28(4)	59	II	33(5)<br>25(4)	58	Improved definition for III Round (**urolithiasis with sepsis**)	48.8(4)<br>30.2(5)	79	Accepted with modifications for redundant items for IV Round	25.8(4)<br>38.7(5)	64.5	**Cancelled**

	**Fournier's gangrene**	62(5)<br>28(4)	90	included									
	**Toxic mega-colon**	60(5)<br>28(4)	88	included									
	**Anastomotic f istula**	48(5)<br>28(4)	76	included									
	**Necrotizing fascitis**	68(5)<br>20(4)	>90	included									
	**Ruptured tubo-ovarian abscess with septic shock**	28(5)<br>34(4)	62	II Round	47(5)<br>23(4)	70	III Round	37.2 (Ls 4)<br>34.9 (Ls 5)	72.1	Included after dis-cussion at WSES Congress 2022			
	**Complicated appendicitis (necrotic, ap-pendicular abscess, per-forated, pres-ence of fecol-ith) associat-ed with local/ generalized peritonitis**	-	-	-	-	-	III Round	27.9 (Ls 4)<br>48.8 (Ls 5)	76.7	Included			

	**Complicated diverticulitis (with local/ generalized peritonitis and sign of septic shock- Hinchey III-IV)**	31(5)<br>40(4)	71	II Rounds	-	-	III Round	25.6 (Ls 4)<br>55.8 (Ls 5)	81.4	Included			
	**Incomplete abortion**	34(5)<br>40(4)	74	II Round	52 (5)<br>29(4)	81	includ-ed	-	-	-			
	**Perianal ab-scess with septic shock**	-	-	-	51(5)<br>23(4)	74	III Round	27.9 (Ls 4)<br>48.8 (Ls 5)	76.7	Included			
	Subdural hematoma	51(5)<br>17(4)	68	II Round	-	-	III Round	34.9 (Ls 4)<br>34.9 (Ls 5)	69.8	Can-celled			
	**Pneumotho-rax**	45(5)<br>40(4)	85	included	-	-	-	-	-	-			

	**Heart-lungs transplants**	65(5)<br>17(4)	82	Included	-	-	-	-		Cancelled after discussion at WSES congress (2022)			
**YELLOW-surgery within 3/6 hrs**	**Contaminated open fractures**	-	-	-	54(5)<br>25(4)	79	Included						
	**Spinal cord compression with cauda equina syndrome**	45(5)<br>43(4)	88	Included	-	-	-						
	Increasing intracranial pressure	-	-	-	-	-	-	27.9 (Ls 4)<br>44.2 (Ls 5)	72.1	Cancelled			
**Patients with sepsis**	**Fournier's gangrene**	-	-	-	68(5)<br>11(4)	79	Included	-	-				
	**Toxic megacolon**	-	-	-	60(5)<br>29(4)	89	Included	-	-				

	Intestinal anastomotic fi stula/insuffi-ciency with sepsis	-	-	-	33(5)<br>35(4)	68	III Round	34.9 (Ls 4)<br>37.2 (Ls 5)	72.1	Can-celled			
	**Necrotizing fascitis**	-	-	-	64(5)<br>15(4)	79	Includ-ed	-	-				
	**GI perfora-tion**	-	-	-	76.5(5)<br>11.8(4)	88.3	Includ-ed						
	**Ruptured tubo-ovarian abscess**	-	-	-	51(5)<br>31(4)	82	Includ-ed						
	**Complicated appendicitis(necrotic, ap-pendicular abscess, per-forated, in presence of fecolith and sign of sepsis)**	51(5)<br>23(4)	74	II Round	41.2(5)<br>31.4(4)	72.6	III Round	37.2 (Ls 4)<br>48.8 (Ls 5)	86	Included			

	**Complicated diverticulitis with sepsis (Hinchey III-IV)**	-	-	-	33(5)<br>31(4)	64	III Round	34.9 (Ls 4)<br>53.5 (Ls 5)	88.4	Included			
	**Urolithiasis with sepsis**	-	-	-	33(5)<br>25(4)	58	III Round	48.8 (Ls 4)<br>30.2 (Ls 5)	79	Included			
	**Incomplete abortion with sepsis**	-	-	-	52(5)<br>29(4)	81	Includ-ed	-	-	-			
	**Perianal ab-scess with sepsis**	28(5)<br>34(4)	62	II Round	39(5)<br>33(4)	72	III Round	53.5 (Ls 4)<br>30.2 (Ls 5)	83.7	Included			
	Intraperitoneal bladder rup-ture	-	-	-	37(5)<br>35(4)	72	III Round	25.6 (Ls 4)<br>41.9 (Ls 5)	67.5	**Can-celled**			
	**Compartment syndrome (any districts)**	-	-	-	66(5)<br>15(4)	81	Includ-ed	-	-	-			

	Foreign body with obstruction (including endoscopy)	-	-	-	29(5)<br>35(4)	64	III Round	16.3 (Ls 4)<br>44.2 (Ls 5)	60.5	**Cancelled**			
	**Liver transplant**	54(5)<br>25(4)	79	Included	-	-				Cancelled after discussion at WSES World Congress 2022			
	Embolization of splenic post-traumatic pseudoaneurysm	-	-	-	41(5)<br>31(4)	72	III Round	23.3 (Ls 4)<br>32.6 (Ls 5)	55.9	**Cancelled**			

**GREEN -surgery within 12 hrs**	Bowel obstruction	45(5)<br>17(4)	62	II Round	37(5)<br>29(4)	66	Improved definition for III Round and moved to Blue class						
	Perianal abscess	37(5)<br>34(4)	71	II Round	41(5)<br>23(4)	64	Moved to Blue class						

	Cholecystitis	34(5)<br>23(4)	57	II Round	35(5)<br>25(4)	60	Moved to Blue class						
	**Appendicitis**	45(5)<br>28(4)	73	II Round	62(5)<br>21(4)	83	Includ-ed						
	**Thoracic empyema**	37(5)<br>40(4)	77	included	-	-	-						
	Incarcerated hernia with obstruction	40(5)<br>20(4)	60	II Round	55(5)<br>18(4)	73	III Round	32.6 (Ls 4)<br>30.2 (Ls 5)	62.8	Moved to Blue class for IV Round			
	Urinary fistula	23(5)<br>23(4)	46	II Round	39(5)<br>22(4)	61	**Can-celled**	-					
	Hydronephro-sis	34(5)<br>28(4)	62	II Round	31(5)<br>25(4)	56	III Round	20.9 (Ls 4)<br>30.2 (Ls 5)	51.1	**Can-celled**			

	Urolithiasis with acute kidney injury	34(5) 28(4)	62	II Round	31(5) 25(4)	56	III Round	27.9 (Ls 4) 44.2 (Ls 5)	72.1	**Can-celled**			
	Kidney trans-plant	42(5) 28(4)	70	II Round	41(5) 25(4)	66	III Round	18.6 (Ls 4) 27.9 (Ls 5)	46.5	**Can-celled**			
	Foreign body without ob-struction	37(5) 31(4)	68	II Round	23(5) 18(4)	41	**Can-celled**						
**BLUE CODE-surgery within 24-48hrs**	**2nd look la-parotomy**	63(5) 23(4)	86	Included									
	**Cholecystec-tomy after ERCP for stones migra-tion or recur-rent biliary colic**	51(5) 25(4)	76	Included									

	Bowel/intestinal obstruction after medical treatment failure	-	-	-	-	-	III Round(moved from green to blue class)	27.9 (Ls 4)<br>41.9 (Ls 5)	69.8	IV Round	18.8(4)<br>31.3(5)	50.1	**Cancelled**
	**Perianal abscess**	-	-	-	-	-	III Round(moved from green to blue class)	34.9 (Ls 4)<br>37.2 (Ls 5)	72.1	Included after discussion at WSES congress 2022			

	**Cholecystitis**	-	-	-	-	-	III Round(moved from green to blue class)	23.3 (Ls 4)<br>55.8 (Ls 5)	79.1	Included			
	Urinary fistula	-	-	-	-	-	III Round(moved from green to blue class)	11.6 (Ls 4)<br>37.2 (Ls 5)	48.8	**Can-celled**			

	Foreign body without obstruction	-	-	-	-	-	III (moved from green to blue class)	16.3 (Ls 4)<br>53.5 (Ls 5)	69.8	**Cancelled**			
	Acute appendicitis without peritoneal fluid	45(5)<br>20(4)	65	II Round	60(5)<br>14(4)	74	III round	14 (Ls 4)<br>51.2 (Ls 5)	65.2	**Cancelled**			
	Amputation for osteomyelitis	31(5)<br>34(4)	65	II Round	58(5)<br>12(4)	70	III Round	32.6 (Ls 4)<br>37.2 (Ls 5)	69.8	**Cancelled**			
	A-V fistulae for hemodialysis	31(5)<br>25(4)	56	II Round	54(5)<br>16(4)	70	III Round	23 (Ls 4)<br>27 (Ls 5)	50	**Cancelled**			
	Symptomatic carotid artery stenosis, TIA, STROKE in evolution	37(5)<br>25(4)	62	II Round	37(5)<br>30(4)	67	**Cancelled**						

	Symptomatic AAA after medical treatment failure	42(5)<br>25(4)	67	II Round	41.2(5)<br>29.4(4)	70.6	**Can-celled-ed**						
	Symptomatic aortic dissection type B	45(5)<br>25(4)	70	II Round	41(5)<br>18 (4)	59	**Can-celled**						
	Bones fracture/complicated bones fractures (displaced/complex)	37(5)<br>31(4)	68	II Round	43(5)<br>25(4)	68	III Round	30.2 (Ls 4)<br>34.9 (Ls 5)	65.1	**Can-celled**			
	**Pelvic trauma fixation**	28(5)<br>43(4)	71	II Round	53(5)<br>25(4)	78	includ-ed						
	Maxillofacial fractures	43(5)<br>25(4)	68	II Round	49(5)<br>23(4)	72	III Round	18.6 (Ls 4)<br>39.5 (Ls 5)	58.1	**Can-celled**			
	Urolithiasis	25(5)<br>37(4)	62	II Round	37(5)<br>25(4)	62	**Can-celled**						

	Incarcerated AW hernia with obstruction	-	-	-	-	-	-	-	-	-	28.1(4)<br>31.3(5)	59.4	**Cancelled**
**ORGANIZATIVE NEED**	**Elective postponed interventions**	37(5)<br>28(4)	65	II Round	54(5)<br>23(4)	77	Included						
	**Diagnostic biopsy/laparoscopy**	31(5)<br>31(4)	62	II Round	58(5)<br>17(4)	75	Included						

Table 5: Summary of all rounds voting results (CCA: Collective Consensus Agreement)
